# Associations of Infant Nutrition with Insulin Resistance Measures in Early Adulthood: Evidence from the Barry-Caerphilly Growth (BCG) Study

**DOI:** 10.1371/journal.pone.0034161

**Published:** 2012-03-27

**Authors:** Dylan M. Williams, Richard M. Martin, George Davey Smith, K. G. M. M. Alberti, Yoav Ben-Shlomo, Anne McCarthy

**Affiliations:** 1 School of Social and Community Medicine, University of Bristol, Bristol, United Kingdom; 2 Department of Endocrinology and Metabolism, St Mary's Hospital, Imperial College, London, United Kingdom; 3 Child Health Epidemiology Division, Health Research Board, Dublin, Ireland; German Diabetes Center, Leibniz Center for Diabetes Research at Heinrich Heine University Duesseldorf, Germany

## Abstract

**Background:**

Previous studies suggest that over-nutrition in early infancy may programme long-term susceptibility to insulin resistance.

**Objective:**

To assess the association of breast milk and quantity of infant formula and cows' milk intake during infancy with insulin resistance measures in early adulthood.

**Design:**

Long-term follow-up of the Barry Caerphilly Growth cohort, into which mothers and their offspring had originally been randomly assigned, between 1972–1974, to receive milk supplementation or not. Participants were the offspring, aged 23–27 years at follow-up (n = 679). Breastfeeding and formula/cows' milk intake was recorded prospectively by nurses. The main outcomes were insulin sensitivity (ISI_0_) and insulin secretion (CIR_30_).

**Results:**

573 (84%) individuals had valid glucose and insulin results and complete covariate information. There was little evidence of associations of breastfeeding versus any formula/cows' milk feeding or of increasing quartiles of formula/cows' milk consumption during infancy (<3 months) with any outcome measure in young adulthood. In fully adjusted models, the differences in outcomes between breastfeeding versus formula/cows' milk feeding at 3 months were: fasting glucose (−0.07 mmol/l; 95% CI: −0.19, 0.05); fasting insulin (8.0%; −8.7, 27.6); ISI_0_ (−6.1%; −11.3, 12.1) and CIR_30_ (3.8%; −19.0, 32.8). There was also little evidence that increasing intakes of formula/cows' milk at 3 months were associated with fasting glucose (increase per quartile of formula/cows' milk intake = 0.00 mmol/l; −0.03, 0.03); fasting insulin (0.8%; −3.2, 5.1); ISI _0_ (−0.9%; −5.1, 3.5) and CIR_30_ (−2.6%; −8.4, 3.6).

**Conclusions:**

We found no evidence that increasing consumption of formula/cows' milk in early infancy was associated with insulin resistance in young adulthood.

## Introduction

Insulin resistance is an important precursor of the onset of type-2 diabetes [1] and a key component of the metabolic syndrome [2], a constellation of adiposity-related risk factors for cardiovascular disease (CVD). There is emerging evidence that early nutrition (both prenatal and throughout infancy) may program physiological function [3] and influence several metabolic traits in later life [2], [4], [5]. Breastfeeding has been inversely associated with CVD risk factors such as blood pressure [6], [7], obesity [8], [9] and adverse cholesterol profiles [10] in later life.

Previous prospective evidence has also suggested inverse associations of breastfeeding with insulin resistance in childhood [4] and adulthood [11], and with type-2 diabetes [12]. However, such associations have not been observed universally [7]. A meta-analysis of published observational studies suggested that breastfeeding was associated with a 39% reduction in risk of type-2 diabetes in adulthood compared with having been formula-fed, and a modest inverse association of breastfeeding with circulating insulin levels in infancy [13]. There were relatively few studies that could be included in the meta-analysis and confounding and publication bias were important concerns. In addition, there was no strong evidence for associations of breastfeeding with fasting insulin in childhood or adulthood, or for associations with fasting glucose measures at any stage of the life-course, although most studies could not examine more sophisticated measures of insulin metabolism.

Previously, Martin *et al* [14] reported a positive association of quantity of formula milk consumed in early infancy with blood pressure levels and body mass index (BMI) in young adults (23–27 years old) in the Barry Caerphilly Growth (BCG) study. Here we investigate the hypothesis that formula and cows' milk intake in early infancy is associated with a reduction in insulin sensitivity and/or an increase in insulin secretion measures in young adulthood.

## Methods

### Participants

The Barry Caerphilly Growth (BCG) cohort study is a long-term follow-up of a randomized controlled trial of 1,163 infants born consecutively between March 1972 and October 1974 in two populations from the towns of Barry and Caerphilly in South Wales [15]. The original aim was to investigate the effects of free milk supplements [through the provision of tokens equating to a half-pint (284 ml) of milk per day] for pregnant women and their infants on birth weight and subsequent childhood growth up to 5 years of age compared to offspring of a control group who received no free milk supplementation. At the end of the study, 951 individuals were seen at five years of age (82% of those born). The provision of milk tokens led to a non-significant increase in birth weight compared with unsupplemented control subjects (in keeping with the results of other studies of this issue in similar populations) and had no effect on height, weight, or skinfold thickness at 5 years [15]. We have therefore treated the study population as a single cohort for the current analysis, with intervention arm included as a covariate.

A follow-up of the 951 participants who had completed the original trial at 5 years was conducted between 1997 and 1999. Subjects were traced through contact with their parents or by invitation sent on our behalf by their local health authority, identified by tracing and flagging the subjects with the National Health Service Central Register [16]. 679 (71%) participants attended the follow-up clinic. Ethical approval for the follow-up study was granted by the Bro Taf Health Authority Local Research Ethics Committee. Informed written consent was obtained from all participants.

### Measurements

#### The original BCG study

Infant formula milk ( dried full-fat cows' milk reconstituted by dilution in water) [17] and cows' milk intake were recorded by research nurses in the original phase of the study during home visits undertaken around 10 days, 6 weeks, and 3, 6, 9 and 12 months from birth. Specific questions were asked about consumption per day of total formula milk and cows' milk (in oz), and whether or not (and when) the infant had been introduced to solids (mixed feeding) [15]. We chose *a priori* to analyse the total infant formula milk and cows' milk intake at 10 days, 6 weeks and 3 months from birth in association with adult insulin measures to allow comparisons of effects from three sensitive periods of early development before a significant proportion of the cohort had been weaned. Information about breastfeeding was not collected directly by the nurses, but we inferred breastfeeding status at 10 days, 6 weeks and 3 months on the basis of infants having no record of intake of formula milk or cows' milk at each of the time periods. However, infants classified as breastfed may or may not have had semi-solid foods introduced (see Table 1 for distributions of solids/semisolids at each age-group by whether breastfed or fed formula/cow's milk). Birth weights for all participants and gestational age for 906 subjects (data missing for 45 individuals) were extracted from hospital records. The social class of the participants' fathers was derived from questionnaires administered when infants were aged 18 months.

**Table 1:** Comparison of baseline characteristics of participants eligible for the final analysis, by gender (mean and SD, unless stated as median and IQR, or %).

	Males (N = 313)	Females (N = 260)	Total (N = 573)
Birth weight (kg)*	3.4±0.5	3.3±0.5	3.4±0.5
Formula/cows' milk intake (ml)∧			
*10 d total*	489.9±283.6	470.7±266.0	481.3±276.2
formula	489.8±283.9	470.7±267.0	481.2±276.3
cows'	0.2±3.2	0.0±0.0	0.1±2.4
*6 wk total*	686.0±292.8	645.3±282.3	667.6±288.6
Formula	679.1±295.8	641.6±286.8	662.0±292.1
cows'	7.0±57.3	3.8±38.1	5.5±49.5
*3 mo total* *	795.6±263.5	689.3±265.3	720.8±265.7
Formula	712.3±296.9	670.7±283.7	693.4±291.4
cows'	34.8±148.0	18.5±108.3	27.4±131.6
% fed infant formula			
10 d	80.5	81.5	80.3
6 wk	89.5	87.3	88.5
3 mo	90.4	89.2	89.9
% fed cows' milk			
10 d	0.3	0.0	0.2
6 wk	4.5	3.1	3.8
3 mo	8.9	3.8	6.6
% breastfed			
10 d	18.9	18.8	18.8
6 wk	9.1	11.7	10.3
3 mo	5.5	8.1	6.7
% fed semi-solids/solids			
10 d	16.4	12.7	14.7
6 wk*	82.0	70.8	76.9
3 mo	92.9	94.9	93.8
% Father's social class			
I/II	19.8	22.3	20.9
III	61.3	51.9	57.1
IV/V	18.8	25.8	22.0
Age at follow-up (years)	25±0.8	25±0.8	25±0.8
Smoking pack years (median & IQR)	1.5 (0, 7.1)	0 (0, 6.3)	0.6 (0, 6.8)
Alcohol (median units/week & IQR)*	23 (14, 32)	10 (4, 17)	16 (6, 28)
BMI (kg/m^2^)	25.0 (15.8, 41.8)	25.5 (17.7, 47.9)	25.2 (15.8, 47.9)
% BMI >25 kg/m^2^	45.8	41.6	43.9
Waist Circumference (cm)*	84.6±9.5	77.5±13.2	81.4±11.8
Fasting Glucose (mg/dl)*	84.7±6.4	79.9±6.4	82.4±6.9
Fasting Insulin (µU/ml)† *	6.4±0.5	7.2±0.5	6.8±0.5
ISI_0_ † 1	18.4±0.5	17.4±0.5	18.0±0.5
CIR_30_ † 2 *	0.66±0.74	0.87±0.75	0.75±0.76

SD = Standard deviation; IQR = Interquartile Range; BMI = Body mass index.

1 Insulin Sensitivity Index whilst fasting = 104/(I0×G0).

2 Corrected Insulin Response at 30 minutes = 100×I30/(G30×(G30−70).

† Geometric means and log SD values.

* Difference in means by gender observed (two sample t-test), all p<0.02.

∧ N for 10 d intake = 568 (312 males, 256 females); N for 6 wk intake = 566 (309 males, 257 females); N for 3 m intake = 569 (310 males, 259 females).

#### Follow-up in adulthood

Subjects completed questionnaires in adulthood about lifetime smoking habits, weekly alcohol consumption and exercise behavior. During research clinic visits, height (to the last complete millimeter), weight (to the nearest kg) and waist circumference, measured at the narrowest point between the costal line and the iliac crest (to the last complete 0.1 cm), were each measured twice and the mean values used in the analysis. Subjects attended the research clinics in the morning after an overnight fast and blood samples were taken for fasting plasma glucose and insulin concentrations. Blood sampling was repeated at 30 minutes and 2 hours after administration of a 75 g oral glucose tolerance test (OGTT). Samples were spun at 3,500 revolutions per minute for 30 minutes and immediately stored at −20°C and sent in batch to Newcastle (UK) for biochemical assay. Plasma glucose was measured in duplicate (Beckman Instruments, Palo Alto, Calif., USA). Serum insulin was determined by ELISA using a two-site immunoassay [18], which does not cross-react with proinsulin (Dako Diagnostics Ltd., Cambridgeshire, UK).

We selected as our primary outcomes for insulin sensitivity (insulin sensitivity index- ISI_0_) and secretion (Corrected insulin response- CIR_30_) [19], [20], those indices which best correlated to corresponding values from the euglycaemic clamp in a normoglycaemic population of similar age (mean 29 years) to that of the BCG cohort [21]. These measures were derived as follows:
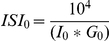


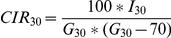
where I_0_ and I_30_ indicate plasma insulin (in µU/ml) when fasting and at 30 minutes after ingestion of 75 g of glucose, respectively, and G_0_ and G_30_ indicate plasma glucose (in mg/dl) when fasting and at 30 minutes after ingestion of 75 g of glucose, respectively. We also included central adiposity (measured by waist circumference) in adulthood as an additional outcome, to examine whether central adiposity could potentially mediate any associations of early infant nutrition with insulin resistance later in life. Participants were excluded from the analysis if they had reportedly eaten within the last 8 hours preceding the OGTT. Participants with high fasting glucose (fasting glucose >160 mg/dl), who may have fasted insufficiently, were excluded (N = 2) [22]. One participant was excluded based on an outlying fasting insulin measure (>10 SD), and 12 participants were excluded because of outlying CIR_30_ measures (>4 SD).

#### Statistical Analysis

We categorised formula and cows' milk intake per day (in oz) at 10 days, 6 weeks and 3 months from birth into quartiles and treated it as an ordinal variable. Participants who were breastfed at 10 days, 6 weeks and 3 months (those who had no recorded intake of formula or cows' milk at these time points) were treated as a separate group. We calculated gender and gestation-standardised z scores for birth weight (between 36–44 weeks), indicating the number of standard deviations each subject's birth weight is in relation to the mean after adjustment for gender and gestational age.

Using detailed questionnaires at follow-up in young adulthood, weekly alcohol consumption in UK standard units (each unit equating to10 milliliters of pure alcohol) was derived by summing recalled intake of beer, spirits, wine and sherry over weekdays and weekends. Pack-years of smoking were derived for subjects to provide an estimate of lifetime exposure, and a 4-group categorical variable was produced with never-smokers as baseline and smokers divided into tertiles by pack years of use. Questions on exercise frequency and duration were used to derive an exercise variable, coded as whether or not subjects regularly participated in an hour or more of strenuous exercise per week [23].

We log transformed outcomes that were positively skewed (fasting insulin, ISI_0_, CIR_30_); hence regression coefficients can be interpreted as a ratio of geometric means rather than a unit change of outcome per unit change of exposure. Associations between exposures and outcomes were investigated using multivariable linear regression. To test whether there was a dose response of formula/cows' milk intake with participant characteristics, *P*-values for trend across quartiles of formula/cows' milk intake (excluding the breastfed category) were computed by entering the quartiles as a continuous (ordinal) variable in the models. We also compared characteristics of participants who were breastfed at 10 days, 6 weeks and 3 months with those who had received any amount of infant formula milk and/or cows' milk (whether or not they were also breastfed). Multivariable models included adjustment for potential confounding factors: model 1 included age at follow-up, gender and intervention group (whether participants were in the control or supplemented groups of the original trial); model 2 included additional adjustment for birth weight z-score, father's social class, and smoking status, alcohol intake and exercise levels in adulthood. The variables adjusted for in model 2 were used as a surrogate measure of maternal influence in promoting a healthy lifestyle as, although these variables are measured in adulthood, mothers who chose to continue breastfeeding (and so feed their infants no or less formula/cows' milk) may also have been more likely to promote beneficial health messages in their children, which subsequently track over time into adult lifestyle. In a sensitivity analysis, we controlled for whether infants had received semi-solids or solids at each age group. We tested whether associations varied by gender using the likelihood ratio test for interaction.

**Figure 1:**
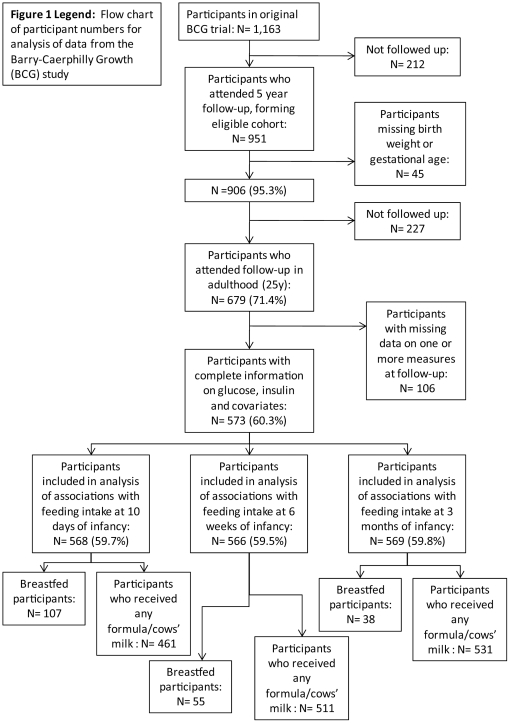
Flow chart of participant numbers for analysis of data from the Barry-Caerphilly Growth (BCG) study.

The analysis was restricted to 573 of 679 individuals with valid glucose and insulin results and complete information on covariates (z-scored birth weight, gender, age at follow-up, adult waist circumference, father's social class, smoking, alcohol and exercise). All regression models were conducted using subjects with complete data on covariates and measures of formula/cows' milk intake (including those breastfed) at each period of recording. Accordingly, models assessing associations of formula/cows' milk intake at 10 days, 6 weeks and 3 months with insulin resistance measures in adulthood were conducted using 568, 566 and 569 subjects, respectively. **Figure 1** shows numbers used in analyses.

## Results

A comparison of baseline characteristics (birth weight, gender, father's social class, intervention group, formula/cows' milk consumption, breastfeeding and whether infants had received semi-solids or solids at each age group) between those followed up and included in the final analysis and those remaining (not followed up or not included in the final dataset) are presented in **Table S1**. No differences in birth weight, gender, father's social class, intervention group assignment or percentages fed semi-solids/solids were observed between the group followed-up (N = 573) and individuals who were not (N = 378). However, there was evidence that the group followed-up had lower intake of formula/cows' milk, and that a higher percentage of this group had been breastfed.

Basic descriptive data on exposures, potential confounding factors and outcomes are shown by gender in **Table 1**. Men had higher mean birth weight and weaning by 6 weeks, and in adulthood had higher alcohol intake, waist circumference and fasting glucose, whilst women had higher mean fasting insulin and insulin sensitivity (CIR_30_).

We compared covariates by whether participants were breastfed or given any formula/cows' milk, alongside covariates by quartile of formula/cows' milk intake at 10 days, 6 weeks and 3 months (**Table 2**). Higher social class and younger age at follow-up were associated with being breastfed at 10 days and 6 weeks, but only younger age at follow up was associated with breastfeeding at 3 months. Birth weight was positively associated with quartile of formula/cows' milk intake at 10 days and 6 weeks but not 3 months, and quantity of formula/cows' milk intake at all three periods was positively associated with being male. No strong associations were seen between quartile of milk intake and the other covariates.

**Table 2:** Adult age and gender adjusted associations of milk intake (breastfed vs. formula/cows' milk fed, or by quartile of formula/cows' milk consumed) at 10 days, 6 weeks and 3 months during infancy of subjects included in final analysis (mean and (95% CI), unless stated as %).

	Birth weight	% Male	% Father's social class			Age at follow-up	% Lifetime smoking (pack years)				Alcohol	% in milk
	(kg)		I/II	III	IV/V		Nil	0.02–4.3	4.4–8.8	9.0–25.3	(units/week)	arm of trial
**Milk intake at 10 d (N = 568)**												
Breastfed (N = 107)	3.6 (3.4, 3.7)	55.1	29.9	50.5	19.6	24.8 (24.6, 25.0)	46.7	22.4	15.9	15.0	37.4 (32.5, 42.3)	49.5
Formula/cows' fed (N = 461)	3.6 (3.5, 3.7)	54.9	18.9	58.6	22.5	25.1 (24.9, 25.3)	45.6	17.4	18.9	18.2	36.6 (32.5, 40.7)	52.7
*P for difference*	0.54	0.95			0.02	0.002				0.37	0.65	0.42
Formula/cows' milk intake quartiles:												
1st (28–511 ml)	3.4 (3.3, 3.5)	52.1	13.0	59.6	27.4	25.1 (24.9, 25.4)	43.2	16.4	21.2	19.2	35.9 (30.9, 40.9)	55.5
2nd (568–597 ml)	3.6 (3.5, 3.8)	50.0	28.0	54.0	18.0	25.1 (24.9, 25.4)	51.0	16.0	19.0	14.0	36.7 (31.4, 42.0)	50.0
3rd (625–682 ml)	3.7 (3.5, 3.8)	54.2	15.3	61.1	23.6	25.0 (24.8, 25.3)	42.4	20.1	16.7	20.8	37.0 (32.1, 41.9)	52.8
4th (710–1023 ml)	3.9 (3.7, 4.0)	69.0	25.4	57.8	16.9	25.1 (24.9, 25.4)	49.3	15.5	18.3	16.9	35.8 (30.6, 41.1)	50.7
*P for trend*	<0.001	0.04			0.05	0.71				0.48	0.57	0.46
**Milk intake at 6 wk (N = 566)**												
Breastfed (N = 55)	3.6 (3.5, 3.8)	48.3	29.3	53.5	17.2	24.7 (24.5, 25.0)	46.6	20.7	17.2	15.5	36.4 (30.6, 42.2)	50.0
Formula/cows' fed (N = 511)	3.6 (3.5, 3.6)	55.3	19.6	57.8	22.6	25.0 (24.8, 25.2)	46.3	17.7	18.1	17.9	36.4 (32.4, 40.5)	51.8
*P for difference*	0.36	0.31			0.08	0.008				0.77	0.98	0.69
Formula/cows' milk intake quartiles:												
1st (28–682 ml)	3.5 (3.4, 3.6)	50.2	21.1	52.1	26.9	25.1 (24.8, 25.3)	49.1	16.4	18.7	15.8	34.9 (30.0, 39.7)	49.7
2nd (710 ml)	3.5 (3.3, 3.6)	50.0	15.0	59.0	26.0	25.0 (24.7, 25.2)	47.0	19.0	20.0	14.0	37.5 (32.3, 42.7)	53.0
3rd (739–852 ml)	3.6 (3.5, 3.8)	59.7	21.4	58.5	20.1	25.0 (24.8, 25.3)	45.9	15.1	18.2	20.8	38.1 (33.4, 42.7)	50.3
4th (909–1563 ml)	3.7 (3.5, 3.8)	64.2	20.5	66.7	12.8	25.1 (24.8, 25.3)	39.7	24.4	14.1	21.8	35.4 (30.2, 40.5)	57.7
*P for trend*	0.002	0.02			0.23	0.95				0.34	0.32	0.45
**Milk intake at 3 mo (N = 569)**												
Breastfed (N = 38)	3.6 (3.4, 3.8)	44.7	29.0	50.0	21.0	24.7 (24.2)	39.5	31.6	13.2	15.8	38.7 (32.2, 45.2)	50.0
Formula/cows' fed (N = 531)	3.6 (3.5, 3.7)	55.3	20.5	57.6	21.8	25.0 (25.0)	46.3	17.3	18.6	17.7	36.8 (32.8, 40.8)	52.4
*P for difference*	0.55	0.21			0.51	0.02				0.38	0.49	0.68
Formula/cows' milk intake quartiles:												
1st (57–682 ml)	3.6 (3.4, 3.7)	49.7	20.7	58.1	21.2	25.1 (24.8, 25.3)	46.9	16.2	20.1	16.8	35.8 (31.1, 40.6)	51.4
2nd (710–796 ml)	3.7 (3.5, 3.8)	53.2	22.2	55.7	22.1	25.0 (24.8, 25.2)	46.2	20.3	16.5	17.1	37.8 (33.1, 42.6)	53.8
3rd (824–909 ml)	3.5 (3.4, 3.7)	60.3	21.5	56.2	22.3	25.1 (24.9, 25.3)	42.2	17.4	22.3	18.2	35.3 (30.5, 40.1)	55.4
4th (938–1421 ml)	3.6, (3.5, 3.8)	64.4	15.1	63.0	21.9	24.9 (24.6, 25.1)	52.1	13.7	13.7	20.6	38.9 (33.7, 44.1)	46.6
*P for trend*	0.67	0.01			0.40	0.85				0.98	0.48	0.64

*P* for trend was calculated across quartiles of formula/cows' milk intake.

In adult-age and gender adjusted models, there was a positive association formula/cows' milk intake at 6 weeks with ISI_0_ (Table 3). There were also inverse associations of formula/cows' milk intake at 6 weeks with fasting insulin and CIR_30_, and a positive association with ISI_0_. These patterns were not, however, consistent with the breastfeeding versus formula/cows' milk comparison: no outcomes were associated with being breastfed compared to being fed formula/cows' milk at any period.

**Table 3:** Adult age and gender adjusted associations of milk intake (breastfed vs. formula/cows' milk fed or by quartile of formula/cows' milk consumed) at 10 days, 6 weeks and 3 months during infancy with fasting glucose, fasting insulin, insulin sensitivity (ISI_0_) and insulin secretion (CIR_30_) measures in later life in subjects included in the final analysis (mean and (95% CI)).

	Fasting Glucose (mg/dl)	Fasting Insulin † (µU/ml)	ISI_0_ 1 †	CIR_30_ 2 †
**Milk intake at 10 d (N = 568)**				
Breastfed (N = 107)	5.00 (4.89, 5.11)	5.6 (4.8, 6.5)	20.0 (17.0, 23.5)	0.52 (0.41, 0.65)
Formula/cows' fed (N = 461)	4.99 (4.89, 5.08)	5.8 (5.1, 6.6)	19.5 (17.0, 22.4)	0.51 (0.42, 0.62)
*P for difference*	0.69	0.62	0.69	0.84
Formula/cows' milk intake quartiles:				
1st (28–511 ml)	4.98 (4.86, 5.10)	5.8 (4.9,6.8)	19.5 (16.4, 23.2)	0.53 (0.42, 0.67)
2nd (568–597 ml)	4.96 (4.83, 5.09)	5.3 (4.5, 6.3)	21.2 (17.7, 25.5)	0.52 (0.40, 0.67)
3rd (625–682 ml)	5.02 (4.91, 5.14)	5.8 (4.9, 6.8)	19.3 (16.3, 22.8)	0.49 (0.39, 0.62)
4th (710–1023 ml)	5.06 (4.93, 5.18)	5.7 (4.8, 6.8)	19.4 (16.2, 23.2)	0.52 (0.40, 0.66)
*P for trend*	0.09	0.90	0.72	0.50
**Milk intake at 6 wk (N = 566)**				
Breastfed (N = 55)	5.07 (4.94, 5.21)	5.2 (4.3, 6.3)	21.2 (17.4, 25.7)	0.47 (0.36, 0.63)
Formula/cows' fed (N = 511)	4.99 (4.90, 5.09)	5.8 (5.1, 6.6)	19.3 (16.9, 22.1)	0.51 (0.42, 0.62)
*P for difference*	0.12	0.13	0.22	0.53
Formula/cows' milk intake quartiles:				
1st (28–682 ml)	4.99 (4.88, 5.09)	6.1 (5.2, 7.1)	18.5 (15.8, 21.7)	0.53 (0.42, 0.66)
2nd (710 ml)	4.97 (4.85, 5.08)	5.8 (4.9, 6.8)	19.6 (16.5, 23.3)	0.55 (0.43, 0.71)
3rd (739–852 ml)	5.01 (4.91, 5.12)	5.5 (4.7, 6.3)	20.4 (17.5, 23.8)	0.49 (0.39, 0.60)
4th (909–1563 ml)	4.95 (4.84, 5.07)	5.4 (4.6, 6.4)	20.8 (17.6, 24.7)	0.43 (0.34, 0.55)
*P for trend*	0.92	0.05	0.06	0.05
**Milk intake at 3 mo (N = 569)**				
Breastfed (N = 38)	5.06 (4.91, 5.21)	5.3 (4.3, 6.5)	21.0 (16.9, 26.2)	0.47 (0.35, 0.64)
Formula/cows' fed (N = 531)	4.99 (4.90, 5.09)	5.8 (5.1, 5.6)	19.5 (17.0, 22.3)	0.50 (0.42, 0.61)
*P for difference*	0.29	0.29	0.39	0.60
Formula/cows' milk intake quartiles:				
1st (57–682 ml)	5.00 (4.90, 5.11)	5.6 (4.8, 6.5)	20.0 (17.1, 23.4)	0.48 (0.39, 0.61)
2nd (710–796 ml)	4.97 (4.86, 5.08)	5.6 (4.8, 6.5)	20.3 (17.3, 23.7)	0.52 (0.42, 0.65)
3rd (824–909 ml)	4.99 (4.89, 5.10)	5.8 (5.0, 6.7)	19.4 (16.6, 22.7)	0.47 (0.38, 0.59)
4th (938–1421 ml)	5.02 (4.90, 5.14)	5.8 (4.9, 6.8)	19.2 (16.2, 22.9)	0.45 (0.35, 0.58)
*P for trend*	0.82	0.51	0.51	0.43

*P*-values are adjusted for age at follow-up. *P* for trend was calculated across quartiles of formula/cows' milk intake.

1 Insulin Sensitivity Index whilst fasting = 10^4^/(I_0_×G_0_).

2 Corrected Insulin Response at 30 minutes = 100×I_30_/(G_30_×(G_30_−70).

† Geometric means with 95% CI.

**Table 4** shows multivariable associations of quartile of infant formula/cows' milk intake at 10 days, 6 weeks and 3 months of infancy with insulin resistance measures in adulthood. In model 1, there were inverse associations of intake at 6 weeks with insulin and CIR_30_, and also a positive association with ISI_0_. However, these attenuated to the null following further adjustment for potential confounders in model 2 (all *P≥*0.09). No other associations were observed between outcomes and the feeding variables recorded at 10 days or 3 months.

**Table 4:** Multivariable regression analyses showing changes in outcomes (and 95% confidence intervals) at follow-up (23–27 y) per quartile of formula/cow's milk intake at 10 days, 6 weeks and 3 months during infancy*.

	10 days (N = 461)			6 weeks (N = 511)			3 months (N = 531)		
	Mean difference per quartile of intake	95% CI	*P*	Mean difference per quartile of intake	95% CI	*P*	Mean difference per quartile of intake	95% CI	*P*
**Fasting Glucose (mmol/l)**
Model 1	0.03	(−0.003, 0.06)	0.08	−0.002	(−0.03, 0.03)	0.88	0.00	(−0.03,0.03)	0.80
Model 2	0.02	(−0.01, 0.06)	0.18	0.00	(−0.03,0.02)	0.75	0.00	(−0.03,0.03)	0.83
**Fasting Insulin** †
Model 1	1.00	(0.96, 1.05)	0.88	0.96	(0.92,1.00)	0.04	1.01	(0.97,1.06)	0.51
Model 2	1.01	(0.97, 1.06)	0.63	0.97	(0.93,1.01)	0.09	1.01	(0.97,1.05)	0.69
**ISI_0_** 1 †									
Model 1	0.99	(0.95, 1.04)	0.70	1.04	(1.00,1.09)	0.05	0.99	(0.94,1.03)	0.50
Model 2	0.98	(0.94, 1.03)	0.51	1.04	(0.99,1.08)	0.10	0.99	(0.95,1.03)	0.68
**CIR_30_** 2 †									
Model 1	0.98	(0.92, 1.04)	0.51	0.94	(0.88,0.99)	0.03	0.98	(0.92,1.04)	0.44
Model 2	0.99	(0.93, 1.06)	0.86	0.95	(0.89,1.01)	0.09	0.97	0.92,1.04)	0.41

Model 1: adjusted for age at follow-up, gender, intervention group.

Model 2: as model 1 plus adjustment for z-score of birth weight, father's social class, lifetime smoking, alcohol intake and exercise.

1 Insulin Sensitivity Index whilst fasting = 10^4^/(I_0_×G_0_).

2 Corrected Insulin Response at 30 minutes = 100×I_30_/(G_30_×(G_30_−70).

† Outcomes were natural-log transformed, and coefficients and confidence intervals represent a change in ratio of geometric means per quartile of formula/cows' milk intake.

* Reference category is those in the lowest quartile of infant formula/cow's milk intake, amongst those who received infant formula/cow's milk.

Multivariable models displaying relative changes in insulin resistance measures of participants who consumed formula/cows' milk compared to those who were breastfed at 10 days, 6 weeks and 3 months during infancy are shown in **Table 5**. There was little evidence for differences in outcomes between feeding groups at any time point in infancy.

**Table 5:** Multivariable regression analyses showing relative changes in outcomes at follow-up (23–27 y) in participants who consumed formula/cow's milk (FF) compared to those who were breastfed (BF) at 10 days, 6 weeks and 3 months during infancy.

	10 days (FF N = 461; BF N = 107 )			6 weeks (FF N = 511; BF N = 55)			3 months (FF N = 531; BF N = 38)		
	Mean difference between groups*	95% CI	*P*	Mean difference between groups*	95% CI	*P*	Mean difference between groups*	95% CI	*P*
**Fasting Glucose (mmol/l)**
Model 1	−0.02	(−0.10,0.06)	0.66	−0.08	(−0.18,0.02)	0.12	−0.07	(−0.19,0.05)	0.28
Model 2	−0.02	(−0.10,0.06)	0.57	−0.08	(−0.18,0.02)	0.13	−0.07	(−0.19,0.05)	0.26
**Fasting Insulin** †
Model 1	1.03	(0.92,1.14)	0.62	1.12	(0.97,1.29)	0.12	1.10	(0.93,1.30)	0.28
Model 2	1.02	(0.92,1.13)	0.73	1.09	(0.95,1.26)	0.21	1.08	(0.91,1.28)	0.37
**ISI_0_** 1 †									
Model 1	0.98	(0.87,1.09)	0.69	0.91	(0.79,1.06)	0.22	0.92	(0.78,1.10)	0.38
Model 2	0.99	(0.88,1.10)	0.81	0.93	(0.80,1.08)	0.33	0.94	(0.79,1.12)	0.49
**CIR_30_** 2 †									
Model 1	0.98	(0.84,1.15)	0.84	1.07	(0.87,1.32)	0.53	1.07	(0.83,1.37)	0.60
Model 2	0.98	(0.84,1.15)	0.85	1.05	(0.85,1.30)	0.63	1.04	(0.81,1.33)	0.78

Model 1: adjusted for age at follow-up, sex, intervention group.

Model 2: as model 1 plus adjustment for z-score of birth weight, father's social class, lifetime smoking, alcohol intake and exercise.

1 Insulin Sensitivity Index whilst fasting = 10^4^/(I_0_×G_0_).

2 Corrected Insulin Response at 30 minutes = 100×I_30_/(G_30_×(G_30_−70).

† Outcomes were natural-log transformed, and coefficients and confidence intervals represent a change in ratio of geometric means between groups.

**Table S2** shows associations of quartile of infant formula/cows' milk intake and a comparison of feeding type with central adiposity (waist circumference) in adulthood. Increasing intake of infant formula/cows' milk at 3 months of infancy was positively associated with waist circumference (relative to those in the lowest quartile of intake), but no other associations were observed.

Since energy intake may differ by gender, we also present associations for quartile of infant formula/cows' milk and insulin resistance measures separately in males and females (**Table S3**). In females (but not males), there was a positive association of infant formula/cows' milk intake at 10 days with fasting insulin, and an inverse association with ISI_0_, after adjustment for potential confounders (both *P* for gender interaction = 0.03) However, all other gender-specific associations were similar to those for the combined sample (consistent with the null hypothesis; all *P* for interaction ≥0.21).

We repeated all analyses adjusting for whether infants had been weaned onto solid or semi-solid foods at 10 days, 6 weeks or 3 months. Results with this additional adjustment were very similar to those in our main analyses (data not shown) suggesting that the associations were not confounded by weaning. We repeated analyses including participants who were excluded based on fasting glucose or insulin measures (N = 15), in the eventuality that these were genuine outliers rather than results from participants who had failed to fast sufficiently before their OGTT. We also recoded these outliers to equal the 99^th^ percentiles of their distributions, for inclusion in a sensitivity analyses. No changes in results from the main models were observed in either case.

## Discussion

We found no evidence of associations between formula and cows' milk intake in infancy and insulin sensitivity (predicted by ISI_0_) or insulin secretion (predicted by CIR_30_) in young adults. This is in contrast to the overall findings of a recent meta-analysis [13] which suggested breastfeeding compared with formula feeding in infancy was associated with lower fasting insulin concentrations in adulthood. In addition, the amount of infant formula/cows' milk intake was not associated with fasting insulin or glucose in early adulthood.

We considered many potential limitations in the interpretation of these results. Selection bias may have occurred if factors relating to participation in the follow-up study in adulthood were in turn associated with both our exposures and outcomes of interest. The baseline characteristics of those participants who were followed up in adulthood were similar to those potentially eligible participants who were not followed up, although formula/cows' milk consumption was lower and breastfeeding more common in participants followed up. However, we see no reason why associations between formula/cows' milk intake and insulin resistance measures in early adulthood would be different amongst participants not followed up.

Information bias regarding our exposure of interest (formula/cows' milk intake) is unlikely to have occurred since data were collected prospectively during nurse visits and our outcomes were measured at research clinic visits over 20 years later. Our measure of breastfeeding is limited because we derived the variable from the absence of recorded formula/cows' milk intake at each time point in infancy, so could not determine the exclusivity of breastfeeding status. However, the comparison of associations between participants who breastfed and those who were given formula/cows' milk is still important for providing comparable results to existing and future papers that also use non-exclusive measures of breastfeeding. Furthermore, controlling for whether infants had received semi-solids or solids at each age group made little difference to the observed associations. Father's social class, recorded when infants were 18 months old, may have poorly represented the social environment that participants were exposed to in infancy and so may not have measured the true level of any socioeconomic influence on the quantity of formula/cows' milk intake and on other factors acting early in life that may be involved in the development of long-term insulin resistance. However, our results changed little after controlling for both father's social class and the inclusion of additional adjustments (lifetime smoking, alcohol intake and exercise), which we have used as proxy measures of both socioeconomic status and attitudes to health behaviours. Nonetheless, even after adjusting for these measures, we cannot rule out the possibility of residual confounding of associations by socioeconomic status. Our results were also independent of birth weight, which may also act as a better proxy of social class at birth than our socioeconomic exposure recorded 18 months after birth [7].

Our study has some important strengths. The hyperinsulinemic euglycaemic clamp is regarded as the “gold standard” measure for determining insulin resistance [24], but is costly, time-consuming and not applicable for large-scale epidemiological studies [25]. Whilst we have also examined fasting insulin and glucose, we were able to derive insulin sensitivity (ISI_0_) and insulin secretion (CIR_30_) as our primary outcomes of interest and these are known to be better correlates to the gold standard than preprandial glucose and insulin levels [21]. A power calculation suggests that our sample size was able to detect a difference in fasting insulin of approximately 1.25 µU/ml (equating to ∼0.28 standard deviations (SD)) between breastfed and formula/cows' milk fed participants at 80% power and 5% significance. The ability to detect less than 0.3 SD difference in fasting insulin is clinically important, given that elevated plasma insulin levels have been positively associated with cardiovascular disease mortality [26].

Another key advantage of our study is that consumption of infant formula milk and cows' milk was quantitatively recorded by visiting research nurses; this contrasts with other studies, which have based analyses on maternal reporting of ever/never breast or bottle-fed status or long-term recall of breastfeeding duration [7], [27]. Thus our results should be less prone to non-differential exposure misclassification, which would otherwise be expected to attenuate any true association to the null.

Owen *et al* [13] conducted a meta-analysis of related studies and reported lower serum glucose and marginally lower serum insulin levels in breastfed infants compared to formula fed infants. They could not exclude the possibility that these results were secondary to both publication bias and uncontrolled or residual confounding. Previously, Martin *et al* had reported a positive association of formula milk intake at 3 months with body mass index (BMI) in the BCG study, and we report a similar association with waist circumference here. No associations with adiposity were found in relation to quantity of formula/cows' milk intake at earlier ages in infancy (10 days and 6 weeks). This observation implies that there is either a particularly sensitive window of post-natal development during which infants are susceptible to metabolic programming through nutritional intake or that the effect is only seen after a sufficient duration of exposure of at least 3 months.

In adults, a close relationship is observed between higher central adiposity and increasing risk of developing insulin resistance [28]. It is not presently clear whether adiposity is a precursor or a result of insulin resistance, but if adiposity does underpin the development of insulin resistance, the observed association between higher formula/cows' milk intake and increased waist circumference in adulthood may carry a concomitant (if marginal) risk for the development of insulin resistance later in the life course. However, we observed correlations of BMI/waist circumference with insulin resistance indices, and although the adiposity measures varied by formula/cows' milk intake at three months, this variation was not reflected in any measure of insulin resistance. It is possible that we did not detect a small effect due to a lack of statistical power or because associations may arise in later adulthood.

Our subjects were exposed to formula milk and its constituents manufactured in the early 1970s which may not be generalisable to breast milk substitutes provided today. Although higher proportions of UK mothers now breastfeed compared with the 1970s, it is estimated that 24% of infants are currently never breastfed and 75% are given infant formula by 6 weeks. Thus, breastfeeding practice is still of major relevance today [29]. Despite our null findings with insulin metabolism, a potential reduction in adult adiposity (as previously reported) would, if translated into reduced morbidity and mortality, have major public health benefits. Further studies should analyse how components of breast milk could plausibly alter the programming of physiological structure and function that influence long-term risks of adiposity and try and resolve if any periods of post-natal development are more or less sensitive to nutritional influences.

Our results suggest that nutrition in the first three months has, if anything, a limited effect on insulin resistance in early adulthood, though we cannot exclude the possibility that these associations may amplify with age, possibly mediated through increased central adiposity. Long term follow-up of large randomised trials of breastfeeding promotion, such as the Promotion of Breastfeeding Intervention Trial (PROBIT) [30], will help resolve whether later effects on insulin-glucose metabolism are seen in an experimental setting, with relatively little influence from confounding factors.

## Supporting Information

Table S1 Comparison of baseline characteristics by subjects who were and were not followed up during the Barry and Caerphilly Growth study, 1972–1974 (mean and SD, unless stated %).
(DOC)

Table S2 Multivariable regression analyses showing changes (and 95% confidence intervals) in waist circumference at follow-up (23–27 y) per quartile of formula/cow's milk intake, and also relative changes in waist circumference of participants who consumed formula/cow's milk (FF) compared to those who were breastfed (BF), at 10 days, 6 weeks and 3 months during infancy.
(DOCX)

Table S3 Multivariable regression analyses showing changes in outcomes (and 95% confidence intervals) at follow-up (23–27 y) per gender-specific quartile of formula/cow's milk intake at 10 days, 6 weeks and 3 months during infancy.
(DOCX)

## References

[1] Lebovitz HE (2006). Insulin resistance - a common link between type 2 diabetes and cardiovascular disease.. Diabetes Obesity & Metabolism.

[2] Ten S, Maclaren N (2004). Insulin resistance syndrome in children.. J Clin Endocrinol Metab.

[3] Lucas A (1998). Programming by Early Nutrition: An Experimental Approach.. J Nutr.

[4] Singhal A, Fewtrell M, Cole TJ, Lucas A (2003). Low nutrient intake and early growth for later insulin stance in adolescents born preterm.. The Lancet.

[5] Singhal A, Cole TJ, Lucas A (2001). Early nutrition in preterm infants and later blood pressure: two cohorts after randomised trials.. The Lancet.

[6] Martin RM, Gunnell D, Davey Smith G (2005). Breastfeeding in Infancy and Blood Pressure in Later Life: Systematic Review and Meta-Analysis.. Am J Epidemiol.

[7] Lawlor DA, Riddoch CJ, Page AS, Andersen LB, Wedderkopp N (2005). Infant feeding and components of the metabolic syndrome: findings from the European Youth Heart Study.. Arch Dis Child.

[8] Owen CG, Martin RM, Whincup PH, Davey Smith G, Cook DG (2005). Effect of Infant Feeding on the Risk of Obesity Across the Life Course: A Quantitative Review of Published Evidence.. Pediatrics.

[9] Mayer-Davis EJ, Rifas-Shiman SL, Zhou L, Hu FB, Colditz GA (2006). Breast-Feeding and Risk for Childhood Obesity.. Diabetes Care.

[10] Owen CG, Whincup PH, Kaye SJ, Martin RM, Davey Smith G (2008). Does initial breastfeeding lead to lower blood cholesterol in adult life? A quantitative review of the evidence.. Am J Clin Nutr.

[11] Ravelli ACJ, van der Meulen JHP, Osmond C, Barker DJP, Bleker OP (2000). Infant feeding and adult glucose tolerance, lipid profile, blood pressure, and obesity.. Arch Dis Child.

[12] Mayer-Davis EJ, Dabelea D, Lamichhane AP, D'Agostino RB, Liese AD (2008). Breast-feeding and type 2 diabetes in the youth of three ethnic groups - The SEARCH for Diabetes in Youth Case-Control Study.. Diabetes Care.

[13] Owen CG, Martin RM, Whincup PH, Davey Smith G, Cook DG (2006). Does breastfeeding influence risk of type 2 diabetes in later life? A quantitative analysis of published evidence.. Am J Clin Nutr.

[14] Martin RM, McCarthy A, Davey Smith G, Davies DP, Ben-Shlomo Y (2003). Infant nutrition and blood pressure in early adulthood: the Barry Caerphilly Growth study.. Am J Clin Nutr.

[15] Elwood PC, Haley TJ, Hughes SJ, Sweetnam PM, Gray OP (1981). Child growth (0–5 years), and the effect of entitlement to a milk supplement.. Arch Dis Child.

[16] Montgomery AA, Ben-Shlomo Y, McCarthy A, Davies D, Elwood P (2000). Birth size and arterial compliance in young adults.. The Lancet.

[17] Department of Health and Social Security (1974). Present day practice in infant feeding.

[18] Andersen L, Dinesen B, Jorgensen PN, Poulsen F, Roder ME (1993). Enzyme immunoassay for intact human insulin in serum or plasma.. Clin Chem.

[19] Sluiter WJ, Erkelens DW, Reitsma WD, Doorenbos H (1976). Glucose tolerance and insulin release, a mathematical approach I. Assay of the beta-cell response after oral glucose loading.. Diabetes.

[20] Sluiter WJ, Erkelens DW, Terpstra P, Reitsma WD, Doorenbos H (1976). Glucose tolerance and insulin release, a mathematical approach. II. Approximation of the peripheral insulin resistance after oral glucose loading.. Diabetes.

[21] Hanson RL, Pratley RE, Bogardus C, Narayan KMV, Roumain JML (2000). Evaluation of Simple Indices of Insulin Sensitivity and Insulin Secretion for Use in Epidemioiogic Studies.. Am J Epidemiol.

[22] (2009). Executive Summary: Standards of Medical Care in Diabetes–2009.. Diabetes Care.

[23] Goodwin RD (2003). Association between physical activity and mental disorders among adults in the United States.. Preventive Medicine.

[24] DeFronzo RA, Tobin JD, Andres R (1979). Glucose clamp technique: a method for quantifying insulin secretion and resistance.. Am J Physiol Gastrointest Liver Physiol.

[25] Bastard JP, Vandernotte JM, Faraj M, Karelis AD, Messier L (2007). Relationship between the hyperinsulinemic-euglycaemic clamp and new simple index assessing insulin sensitivity in overweight andáobese postmenopausal women.. Diabetes & Metabolism.

[26] Hu G, Qiao Q, Tuomilehto J, Eliasson M, Feskens EJ (2004). Plasma insulin and cardiovascular mortality in non-diabetic European men and women: a meta-analysis of data from eleven prospective studies.. Diabetologia.

[27] Fall CHD, Osmond C, Barker DJP, Clark PMS, Hales CN (1995). Fetal and infant growth and cardiovascular risk factors in women.. BMJ.

[28] Lebovitz HE, Banerji MA (2005). Point: Visceral Adiposity Is Causally Related to Insulin Resistance.. Diabetes Care.

[29] Bolling K, Grant C, Hamlyn B, Thornton A (2007). Infant Feeding Survey 2005..

[30] Kramer MS, Matush L, Vanilovich I, Platt RW, Bogdanovich N (2007). Effects of prolonged and exclusive breastfeeding on child height, weight, adiposity, and blood pressure at age 6.5 y: evidence from a large randomized trial.. Am J Clin Nutr.

