# Supplementary material for: Associations of Infant Nutrition with Insulin Resistance Measures in Early Adulthood: Evidence from the Barry-Caerphilly Growth (BCG) Study
Source: PLoS One. 2012 Mar 27;7(3):e34161. doi: 10.1371/journal.pone.0034161 (PMC3313975; doi:10.1371/journal.pone.0034161)
Supplement: Table S1 — Comparison of baseline characteristics by subjects who were and were not followed up during the Barry and Caerphilly Growth study, 1972–1974 (mean and SD, unless stated %). (DOC) [file pone.0034161.s001.doc]

Table S1- Comparison of baseline characteristics by subjects who were and were not followed up during the Barry and Caerphilly Growth study, 1972–1974 (mean and SD, unless stated %)

|  | Group not followed-up | Group followed-up |  |
| --- | --- | --- | --- |
|  | (N= 378) | (N= 573) | *P for difference** |
| Birth weight (kg) | 3.3 ± 0.5 | 3.4 ± 0.5 | 0.06 |
| % Males | 52.4 | 54.6 | 0.50 |
| Father's social class |  |  |  |
| I/II | 16.5 | 20.9 |  |
| III | 59.5 | 57.1 |  |
| IV/V | 24.0 | 22.0 | 0.27 |
| Intervention group (%) |  |  |  |
| Milk tokens | 56.1 | 51.9 |  |
| Control | 43.9 | 48.1 | 0.21 |
| Consumption of formula/cows' milk (ml) |  |  |  |
| 10 d | 519.1 ± 244.2 | 481.3 ± 276.2 | 0.04 |
| 6 wk | 728.9 ± 246.8 | 667.6 ± 288.6 | <0.001 |
| 3 mo | 754.2 ± 248.6 | 720.8 ± 265.7 | 0.05 |
| % breastfed |  |  |  |
| 10 d | 13.9 | 18.8 | 0.05 |
| 6 wk | 5.1 | 10.3 | 0.005 |
| 3 mo | 3.7 | 6.7 | 0.05 |
| % fed semi-solids/solids |  |  |  |
| 10d | 17.7 | 14.9 | 0.31 |
| 6wk | 77.3 | 77.2 | 0.53 |
| 3mo | 93.9 | 93.9 | 0.48 |

SD= Standard deviation

* Difference in means tested by two sample t-test
