# Supplementary material for: Associations of Infant Nutrition with Insulin Resistance Measures in Early Adulthood: Evidence from the Barry-Caerphilly Growth (BCG) Study
Source: PLoS One. 2012 Mar 27;7(3):e34161. doi: 10.1371/journal.pone.0034161 (PMC3313975; doi:10.1371/journal.pone.0034161)
Supplement: Table S2 — Multivariable regression analyses showing changes (and 95% confidence intervals) in waist circumference at follow-up (23–27 y) per quartile of formula/cow's milk intake, and also relative changes in waist circumference of participants who consumed formula/cow's milk (FF) compared to those who were breastfed (BF), at 10 days, 6 weeks and 3 months during infancy. (DOCX) [file pone.0034161.s002.docx]

Table S2- Multivariable regression analyses showing changes (and 95% confidence intervals) in waist circumference at follow-up (23-27y) per quartile of formula/cow’s milk intake, and also relative changes in waist circumference of participants who consumed formula/cow’s milk (FF) compared to those who were breastfed (BF), at 10 days, 6 weeks and 3 months during infancy.

|  | 10 days (N= 568) | | | 6 weeks (N=566) | | | 3 months (N= 569) | | |
| --- | --- | --- | --- | --- | --- | --- | --- | --- | --- |
|  | Mean difference (cm) | 95% CI | *P* | Mean difference (cm) | 95% CI | *P* | Mean difference (cm) | 95% CI | *P* |
| *Per quartile of FF intake* |  |  |  |  |  |  |  |  |  |
| Model 1 | 0.61 | (-0.41,1.62) | 0.24 | -0.40 | (-1.32,0.52) | 0.39 | 1.39 | (0.46,2.31) | 0.003 |
| Model 2 | 0.63 | (-0.42,1.68) | 0.24 | -0.41 | (-1.34,0.51) | 0.38 | 1.16 | (0.25,2.08) | 0.01 |
|  |  |  |  |  |  |  |  |  |  |
| *FF vs. BF* |  |  |  |  |  |  |  |  |  |
| Model 1 | 1.09 | (-1.32,3.50) | 0.38 | 1.11 | (-2.06,4.28) | 0.49 | 0.67 | (-3.10,4.43) | 0.73 |
| Model 2 | 0.63 | (-1.76,3.03) | 0.60 | 0.47 | (-2.68,3.62) | 0.77 | 0.43 | (-3.30,4.16) | 0.82 |

Model 1: adjusted for age at follow-up, gender, intervention group

Model 2: as model 1 plus adjustment for z-score of birth weight, father's social class, lifetime smoking, alcohol intake and exercise
