# Supplementary material for: Associations of Infant Nutrition with Insulin Resistance Measures in Early Adulthood: Evidence from the Barry-Caerphilly Growth (BCG) Study
Source: PLoS One. 2012 Mar 27;7(3):e34161. doi: 10.1371/journal.pone.0034161 (PMC3313975; doi:10.1371/journal.pone.0034161)
Supplement: Table S3 — Multivariable regression analyses showing changes in outcomes (and 95% confidence intervals) at follow-up (23–27 y) per gender-specific quartile of formula/cow's milk intake at 10 days, 6 weeks and 3 months during infancy. (DOCX) [file pone.0034161.s003.docx]

Table S3- Multivariable regression analyses showing changes in outcomes (and 95% confidence intervals) at follow-up (23-27y) per gender-specific quartile of formula/cow’s milk intake at 10 days, 6 weeks and 3 months during infancy.

|  | 10d (N= 568) | | | 6wk (N=566) | | | 3mon (N= 569) | | |
| --- | --- | --- | --- | --- | --- | --- | --- | --- | --- |
|  | Coeff | 95% CI | *P* | Coeff | 95% CI | *P* | Coeff | 95% CI | *P* |
| **Fasting Glucose (mmol/l)** |  |  |  |  |  |  |  |  |  |
| *Model 1* |  |  |  |  |  |  |  |  |  |
| Male | 0.01 | (-0.03,0.05) | 0.61 | -0.01 | (-0.05,0.03) | 0.65 | -0.01 | (-0.05,0.03) | 0.72 |
| Female | 0.05 | (0.002,0.10) | 0.04 | 0.01 | (-0.03,0.05) | 0.69 | 0.02 | (-0.02,0.06) | 0.35 |
| *Model 2* |  |  |  |  |  |  |  |  |  |
| Male | 0.00 | (-0.04,0.05) | 0.87 | -0.01 | (-0.05,0.03) | 0.68 | 0.00 | (-0.04,0.04) | 0.99 |
| Female | 0.04 | (-0.01,0.09) | 0.12 | 0.00 | (-0.04,0.05) | 0.86 | 0.02 | (-0.02,0.06) | 0.40 |
| **Fasting Insulin** † |  |  |  |  |  |  |  |  |  |
| *Model 1* |  |  |  |  |  |  |  |  |  |
| Male | 0.96 | (0.91,1.02) | 0.18 | 0.95 | (0.90,1.004) | 0.07 | 1.00 | (0.95,1.06) | 0.92 |
| Female | 1.06 | (0.99,1.13) | 0.12 | 0.98 | (0.92,1.04) | 0.51 | 1.02 | (0.97,1.08) | 0.42 |
| *Model 2* |  |  |  |  |  |  |  |  |  |
| Male | 0.95 | (0.90,1.01) | 0.11 | 0.96 | (0.91,1.01) | 0.12 | 1.00 | (0.95,1.06) | 0.999 |
| Female | 1.08 | (1.01,1.17) | 0.03 | 0.98 | (0.92,1.04) | 0.49 | 1.03 | (0.97,1.09) | 0.32 |
| **ISI_0_ ^1^** † |  |  |  |  |  |  |  |  |  |
| *Model 1* |  |  |  |  |  |  |  |  |  |
| Male | 1.04 | (0.98,1.10) | 0.22 | 1.05 | (0.996,1.11) | 0.07 | 1.00 | (0.94,1.06) | 0.96 |
| Female | 0.94 | (0.87,1.01) | 0.08 | 1.02 | (0.95,1.09) | 0.57 | 0.97 | (0.92,1.03) | 0.37 |
| *Model 2* |  |  |  |  |  |  |  |  |  |
| Male | 1.05 | (0.99,1.11) | 0.14 | 1.05 | (0.99,1.11) | 0.12 | 1.00 | (0.94,1.06) | 0.996 |
| Female | 0.91 | (0.85,0.99) | 0.02 | 1.02 | (0.96,1.09) | 0.53 | 0.97 | (0.91,1.03) | 0.29 |
| **CIR_30_ ^2^** † |  |  |  |  |  |  |  |  |  |
| *Model 1* |  |  |  |  |  |  |  |  |  |
| Male | 0.98 | (0.90,1.07) | 0.63 | 0.92 | (0.85,0.99) | 0.03 | 0.94 | (0.87,1.02) | 0.14 |
| Female | 0.98 | (0.88,1.08) | 0.63 | 0.96 | (0.88,1.05) | 0.40 | 1.01 | (0.92,1.10) | 0.87 |
| *Model 2* |  |  |  |  |  |  |  |  |  |
| Male | 0.98 | (0.90,1.06) | 0.59 | 0.92 | (0.85,1.00) | 0.06 | 0.93 | (0.86,1.01) | 0.09 |
| Female | 1.00 | (0.90,1.12) | 0.94 | 0.96 | (0.88,1.06) | 0.41 | 0.99 | (0.91,1.08) | 0.85 |

Model 1: adjusted for age at follow-up, gender, intervention group

Model 2: as model 1 plus adjustment for z-score of birth weight, father's social class, lifetime smoking, alcohol intake and exercise

^1^ Insulin Sensitivity Index whilst fasting= 10^4^/(I_0_ x G_0_)

^2^ Corrected Insulin Response at 30 minutes= 100 x I_30_/(G_30_ x (G_30_ –70)

† Outcomes were natural-log transformed, and coefficients and confidence intervals represent a change in ratio of geometric means per quartile of formula/cows’ milk intake
